# An exploration of the solution of direct methanol fuel cell cost effectiveness

**DOI:** 10.3389/fchem.2024.1434996

**Published:** 2024-08-08

**Authors:** Tengyi Wang, Zhiwei Luo, Changsheng Wang, Yang Li, Xi Chen, Yang Tang, Xinsheng Wang, Zhiquan Zhou

**Affiliations:** ^1^ Department of Electronics Science and Technology, Harbin Institute of Technology, Weihai, China; ^2^ Weihai Key Laboratory of Marine Sensors, Weihai, China; ^3^ Harbin Institute of Technology (Weihai) International Microelectronics Center, Weihai, China; ^4^ Shandong Provincial Key Laboratory of Marine Electronic Information and Intelligent Unmanned Systems, Weihai, China; ^5^ Key Laboratory of Cross-Domain Synergy and Comprehensive Support for Unmanned Marine Systems, Ministry of Industry and Information Technology, Weihai, China

**Keywords:** micro direct methanol fuel cell, printed circuit board, current collector, corrosion, cost effectiveness

## Abstract

The work in this paper incorporated printed circuit board (PCB) technology into micro-direct methanol fuel cells (µDMFCs) and conjectured and verified the performance degradation factors of PCB current collectors in µDMFCs by testing different designed configuration µDMFCs. The experiment results showed that all kinds of PCB coating can benefit from the porous stainless-steel plates covering to a great extent. At the end of 48 h discharging, µDMFCs with porous stainless-steel plates between MEA and PCB coating achieved higher performance than those of the direct contacting series. It can be inferred from various types of experimental data that because of stainless-steel porous plate isolating, the impact of corrosion on the surface of the PCB electrode plate was reduced to a certain extent. The corrosion of the electrode plate was slowed down in the µDMFC discharging as a result of the passivation behavior on the iron surface and a decrease in corrosion current. Consequently, the attenuation of the PCB performance was delayed. The conclusion of this work explores a practical direction to enhance the cost-effectiveness of fuel cells, promoting the large-scale application of DMFCs in the future.

## 1 Introduction

A fuel cell is a kind of high-energy-density power source, especially a direct methanol fuel cell (DMFC), which has high portability without the limitation of the hydrogen-holder (Baldauf and Preidel, 1999; Arico et al., 2001; Kamarudin et al., 2007; Kamarudin et al., 2009; Seo and Lee, 2010; Achmad et al., 2011; Taner, 2015; Cao et al., 2018; Zhou et al., 2021; Cao et al., 2022). However, the practical application of fuel cells has always been hindered by the issue of the high usage cost, which, in the form of a low lifespan per power density, restricts their large-scale application. The operation life of most direct methanol fuel cell stacks is shorter than 1 year (80% performance reduction). Although the cost of fuel for power generation is low, the performance degradation of the fuel cell stack leads to high usage costs. The cost of the fuel cell stack includes the cost of MEA for power generation and plates for current transmission, especially electrode plates, which are easy to corrode and make up a significant part of the cost (62% at 40,000 W production per year) (Sgroi et al., 2016). In the past few decades, driven by advancements in commercial fuel cell vehicle low-load catalyst technology, the cost of MEA has been reduced. To address the problem of electrode plate costs, strategies such as either enhancing the lifespan or decreasing the manufacturing cost— in other words, enhancing the cost-effectiveness and large-scale application of fuel cells—can be employed. A long usage lifespan means a high processing cost, while a low cost means a low usage lifespan. The work in this paper aims to find out an optimized balance point and the regulation of PCB technology-based electrode plates between a long lifespan and low cost.

In this paper, three series and six kinds of self-breathing μDMFCs with micro-sized configurations are designed, fabricated, and tested. The cathode and anode were based on printed circuit board (PCB) technology with different metal layers to collect the current. The battery performance, impedance, and corrosion properties of electrodes under different shapes of plates and different surface treatments were tested and compared.

The novel current collectors combined with PCB technology may find a possible balance point between the lifespan and the cost on the DMFC application research, improving the performance and stability of low-cost PCB DMFCs to the level of conventional stainless-steel current collectors. The method proposed in this paper realizes the practical application of PCB technology in the DMFC current collectors, sampling the electronic connections and saving the cost advantages of PCB technology. This exhibits its excellent potential as a candidate for future practical portable power sources.

## 2 Fabrication and assembly

### 2.1 Design and fabrication of the current collectors

**FIGURE 1:**
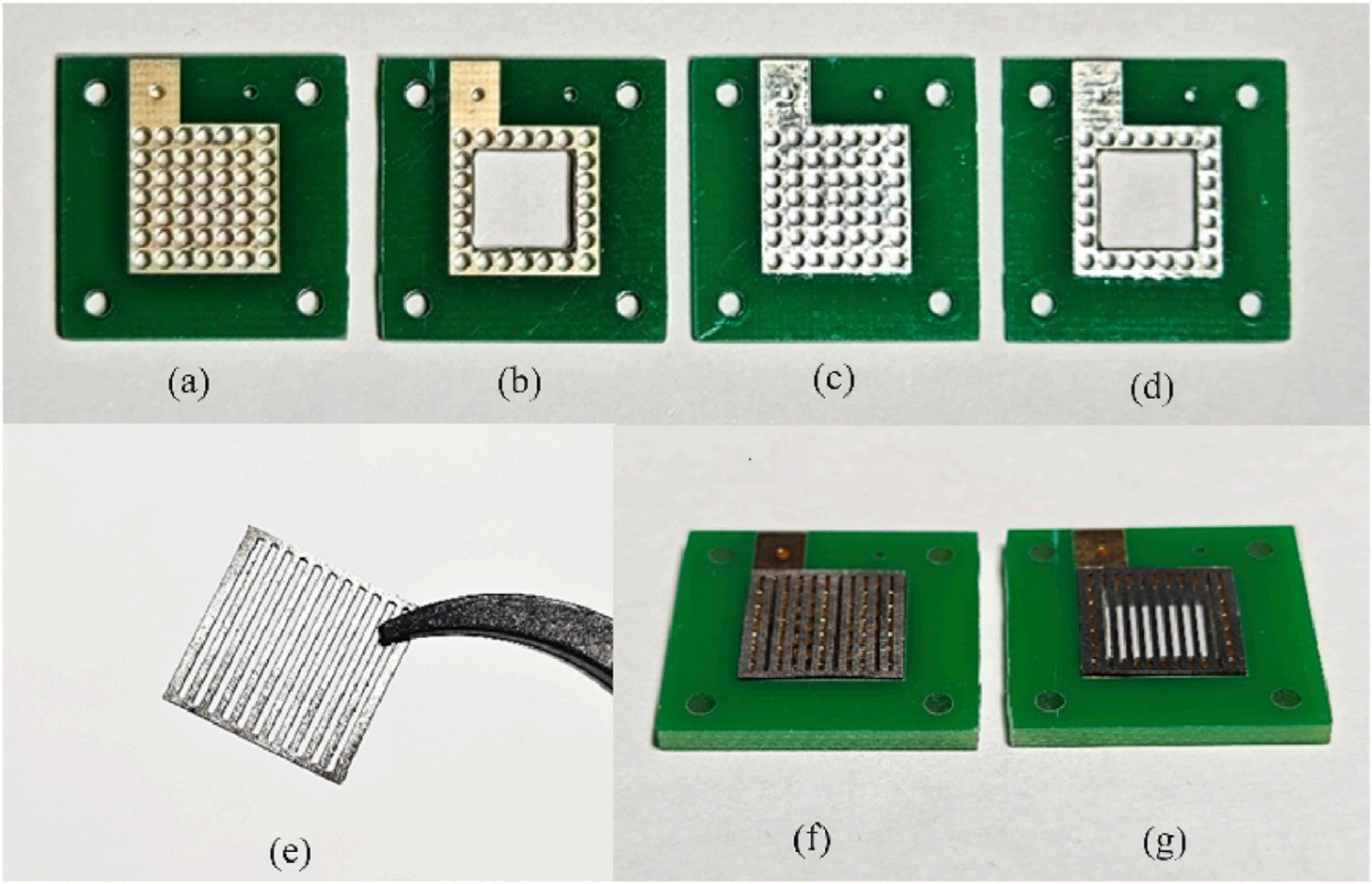
PCB electrodes for the different designed configurations (A,B: 10 µm Au-electroplated Cu surface; C,D: 10 µm Sn-electroplated Cu surface; E: 316L stainless-steel plate; F,G: PCB current collectors with stainless-steel plates).

Both anode and cathode current collectors were manufactured on a PCB with an active area of 1 × 1 cm. Considering the no-water-flooding condition under the scale of micro DMFCs, a perforated pattern with a 300-μm radius hole is designed (Figures 1A, B). The contact areas of the PCB current collector plates were the Au–Cu or Sn–Cu layer based on a fiberglass board. Each plate had 49 patterns arranged as a 7 × 7 square in total. The second configuration of the PCB current collector plates hollowed out a 5 by 5 pattern in the middle, with 24 holes along the edge to facilitate the welding of porous stainless-steel plates.

To evaluate the corrosion resistance of various materials, each type of PCB electrode was treated with two surface treatment methods: 10 μm Au-electroplated Cu surface (Figures 1A, B) and 10 μm Sn-electroplated Cu surface (Figures 1C, D). To explore the corrosion mechanism exploration of PCB coating in DMFC operation, a 300-μm-thick porous 316L stainless-steel plate with up and down parallel openings (a channel width of 600 μm and a rib width of 500 μm, Figure 1E) was designed to barrier and buffer the impact of MEA physical or chemical reactions. These designed 300-μm-thick porous stainless-steel plates were welded to perforated Au- or Sn-coated PCB (Figure 1F) as the current collectors, respectively. In addition, a hollow configuration of PCB current collectors with a square inner hole was designed and tested to assess the impact of corrosion in different areas of PCB current collectors (Figure 1G).

### 2.2 Assembly of the MEA

A carbon-supported catalyst, purchased from Johnson Matthey, Inc., was used for the fabrication of the catalyst layer. The anode catalyst was 60 wt% PtRu (with an atomic ratio of 1:1)/C, and the cathode catalyst was 40 wt% Pt/C. The gas diffusion layers (GDLs) for the cathode electrodes were wet-proofed Toray carbon papers coated with MPLs, which were comprised of Vulcan XC-72 Carbon Black and 10 wt% of PTFE. The anode GDLs were the Toray carbon papers coated with the MPLs, which comprised Vulcan XC-72 Carbon Black and 10 wt% of Nafion ionomer (DuPont). The loading of carbon black was 4 mg cm^-2^ for both the anode and cathode. The catalyst powder and 5 wt% Nafion ionomer solution were ultrasonically mixed in isopropyl alcohol to form a homogeneous catalyst ink. Then, the catalyst ink was sprayed onto the GDLs, and the electrodes were dried for 2 h in a vacuum oven at 80°C. The Nafion content in both the anode and cathode was 20 wt%, and metal loading (PtRu or Pt) was 4 mg cm^-2^ in the anode and 2 mg cm^-2^ in the cathode (Li et al., 2014).

**FIGURE 2:**
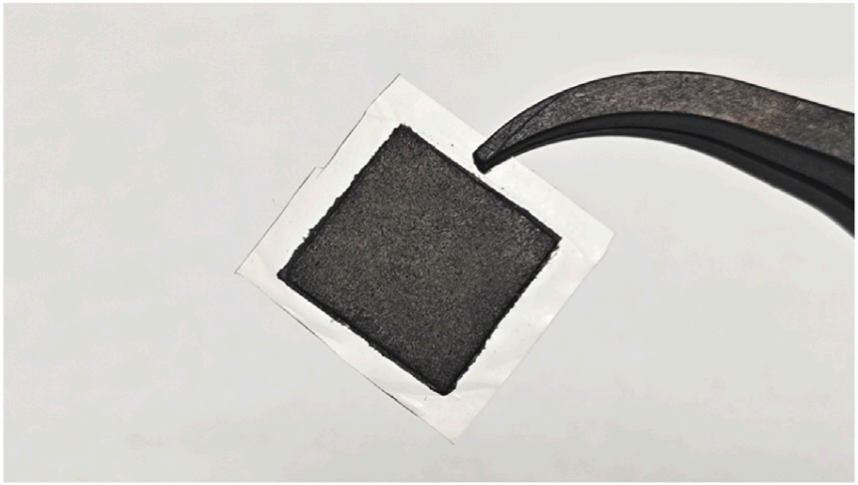
Photograph of the fabricated MEA.

Nafion 117 polymer membranes (DuPont) were used to fabricate MEAs. Before being applied to the electrodes, to eliminate the organic and inorganic contaminants, the membranes were pretreated in 3 wt% H_2_O_2_, deionized water, 3 wt% H_2_SO_4_, and deionized water again for 1 h in each solution in turn. The pretreated Nafion 117 membrane was sandwiched between the anode and cathode, and then, the assembly was hot-pressed under a loading of 18 Mpa for 180 s at 135°C (Liu et al., 2015; Zhao et al., 2009). The prepared MEA has an active area of 10 mm × 10 mm and a thickness of 1 mm, as shown in Figure 2.

### 2.3 Assembly of μDMFCs

The prepared MEA was sandwiched between the anode and cathode current collectors. A polymethylmethacrylate (PMMA) reservoir was fixed to the anode current collector side. In this way, an apparent encapsulation approach was designed and applied to constitute the structures, enhancing the integration of the whole configuration.

In this design, three series of current collectors were designed, and each series contained two surface coatings.

**FIGURE 3:**
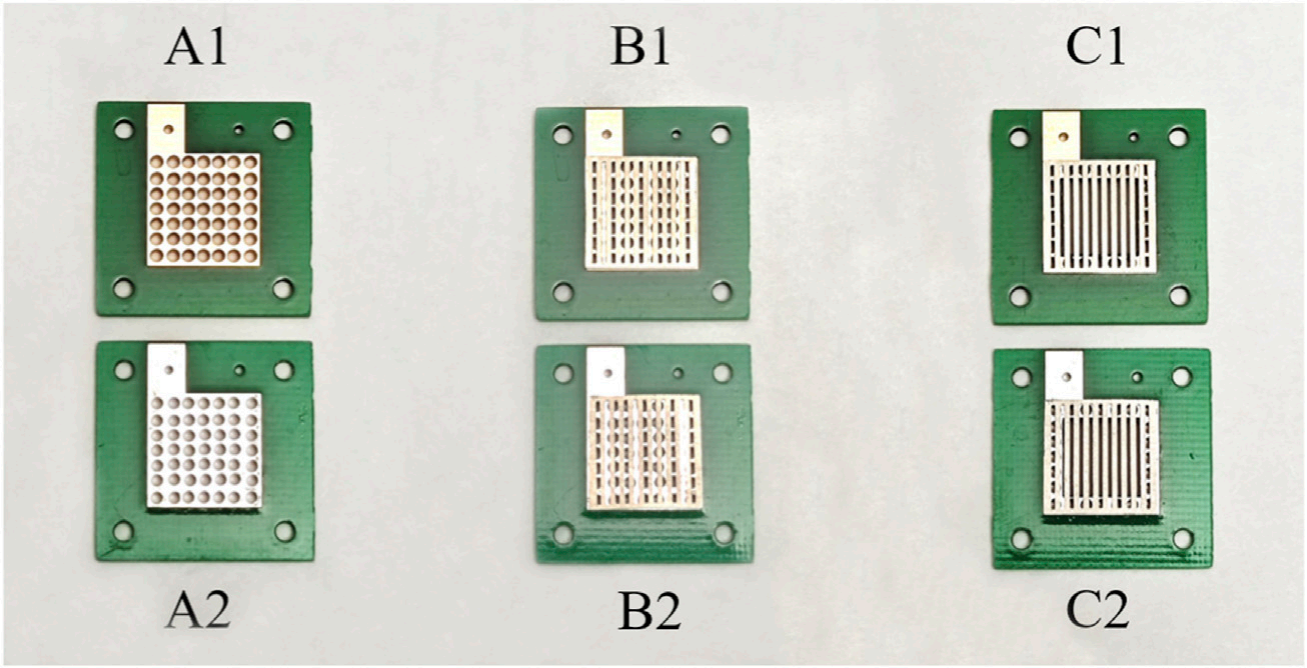
Grouping of different configuration current collectors (A1: PCB collectors of µDMFC; A2: PCB collectors of µDMFC; B1: PCB collectors of µDMFC; B2: PCB collectors of µDMFC; C1: PCB collectors of µDMFC; C2: PCB collectors of µDMFC).

In Group A, two micro-direct methanol fuel cells (μDMFCs) with different surfaces were assembled. Current collectors of cell as shown in Figure 3A1 were both 10 μm Au-electroplated Cu surfaces, and those for cell as shown in Figure 3A2 were both 10 μm Sn-electroplated Cu surfaces.

In Group B, every μDMFC had the same structure with the same serial number as in Group A, but porous 316L stainless-steel plates were added between each PCB electrode and the MEA. As shown in Figures 3B1, B2.

In Group C, every μDMFC had the same structure with the same serial number as in Group B but had hollowed-out PCBs. As shown in Figures 3C1, C2.

**FIGURE 4:**
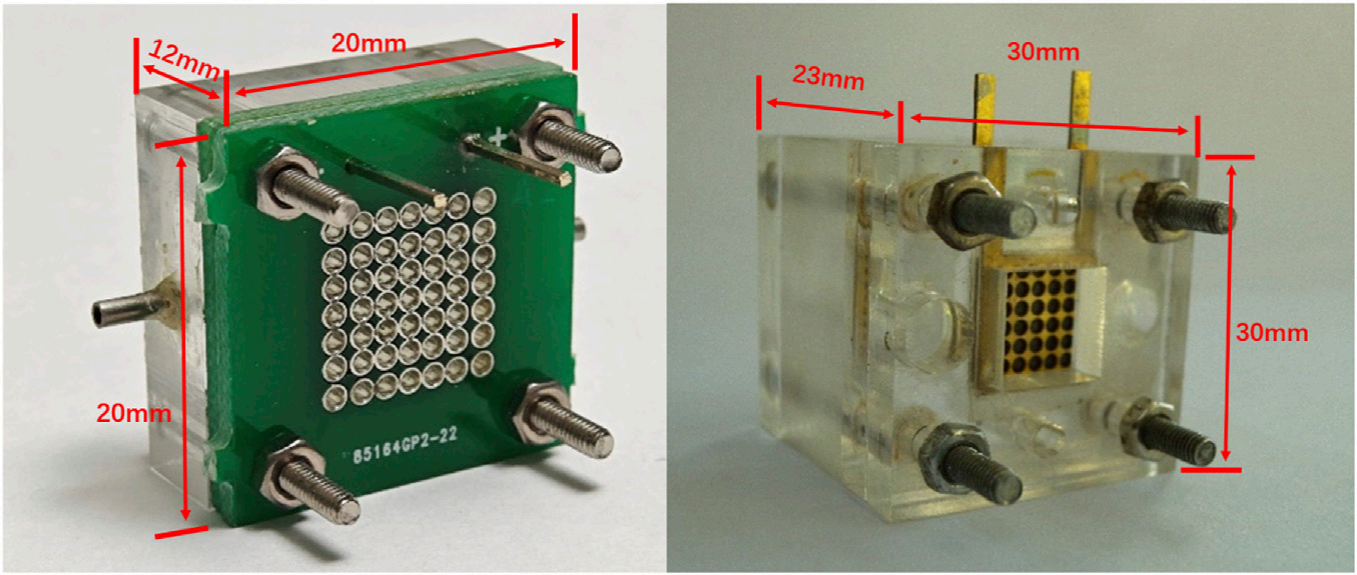
Appearance of the PCB and conventional configuration μDMFC.

This approach will also be an advantage in fuel cell stack studies because of its easy integration. Figure 4 shows the novel micro-sized configuration of the μDMFC, which has a very small dimension of 20 mm × 20 mm × 12 mm.

The characteristics of every μDMFC are shown in Table 1.

**TABLE 1:** Comparison of the structures of the assembled μDMFCs.

	PCB shape	PCB surface	Porous plate
μDMFC A1	49 patterns	(10 μm) AuCu	No
μDMFC A2	49 patterns	(10 μm) Sn–Cu	No
μDMFC B1	49 patterns	(10 μm) Au–Cu	Yes
μDMFC B2	49 patterns	(10 μm) Sn–Cu	Yes
μDMFC C1	Hollowed out	(10 μm) Au–Cu	Yes
μDMFC C2	Hollowed out	(10 μm) Sn–Cu	Yes

Additionally, conventional configuration DMFCs using thickened stainless-steel plates and PMMA fixtures were also assembled for electrode activation before the discharging test and MEA membrane performance testing after the discharge.

## 3 Experiment results and discussion

Before the performance test, the cell was activated by the conventional configuration DMFC fixtures with a 2 M methanol solution at 0.2 V and 60°C until the output current stabilized. After a batch of activations, six MEAs with the same output current were selected to explore the influence of the three series of current collectors.

To evaluate the stability of the µDMFC, the fabricated cell was discharged at a constant voltage of 0.2 V at 25°C. During the 48 h discharging test, the methanol solution in each μDMFC was circulated using the peristaltic pump linked to a 1L 0.2 M methanol tank in order to approximately maintain the concentration of the methanol solution.

**FIGURE 5:**
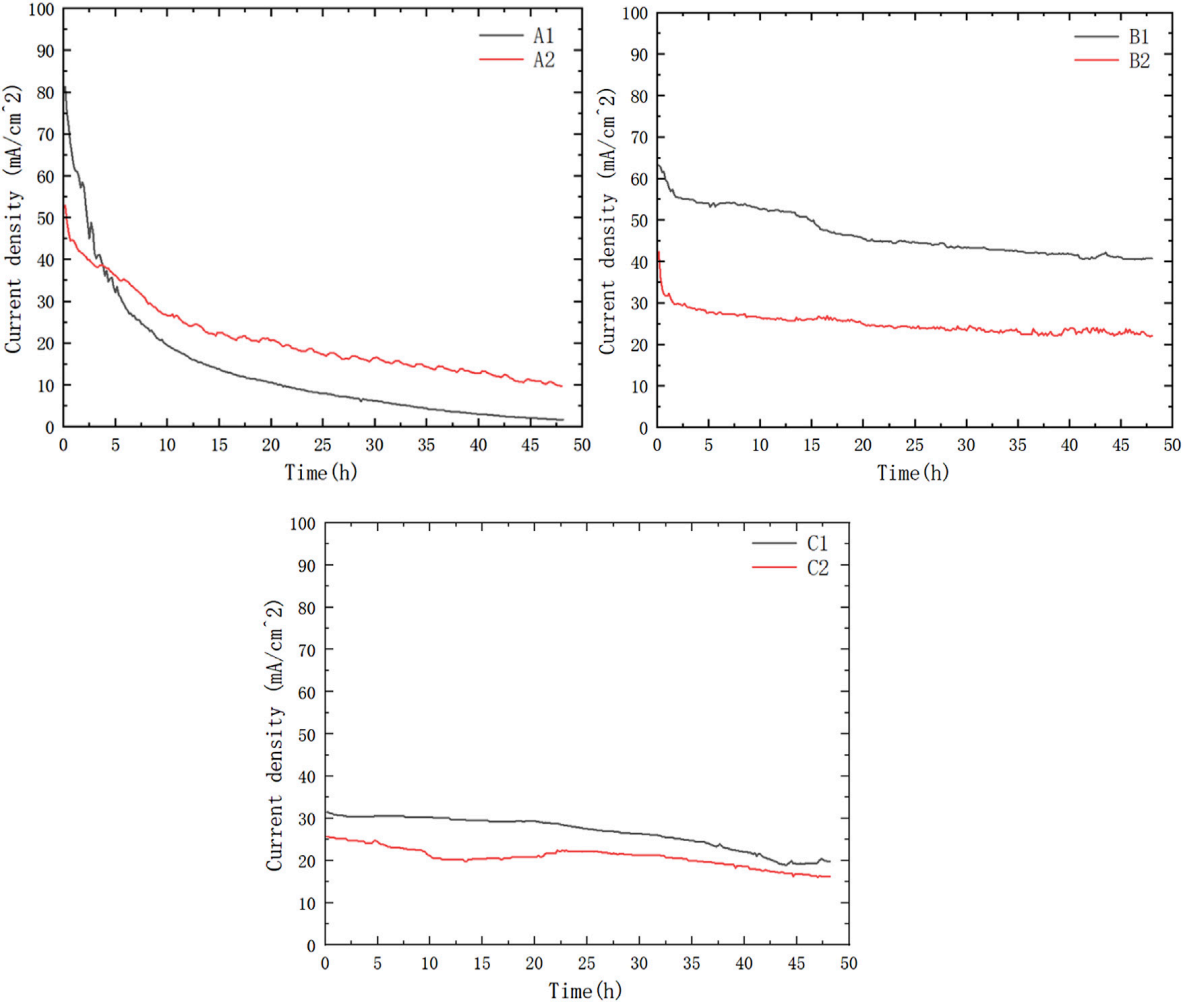
Current density at 0.2 V constant output voltage discharging.

**FIGURE 6:**
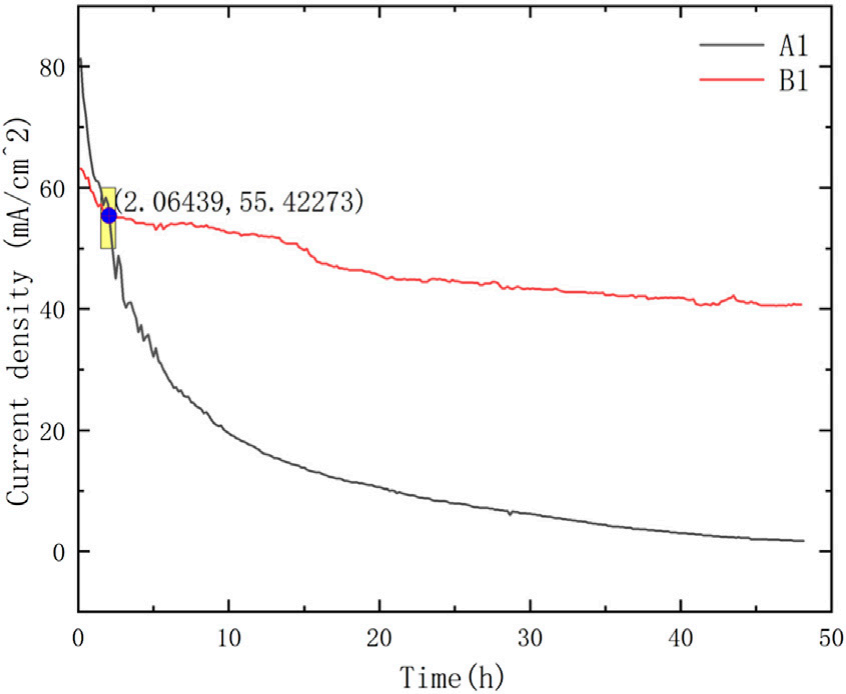
Intersection point of μDMFC A1 and μDMFC B1.

As shown in Figures 5, 6, the current density of Group A was relatively high at the beginning; however, during the 48-h discharging test, the current density of Group A decreased rapidly and was generally lower than that of Group B. Group C showed a lower power density than Group B but showed almost the same level of performance degradation as Group A. The low power density can be attributed to the low packing pressure configuration of the current collectors. The comparable performance degradation may be attributed to using the same porous stainless-steel plate as the current collector in contact with the membrane electrode.

**FIGURE 7:**
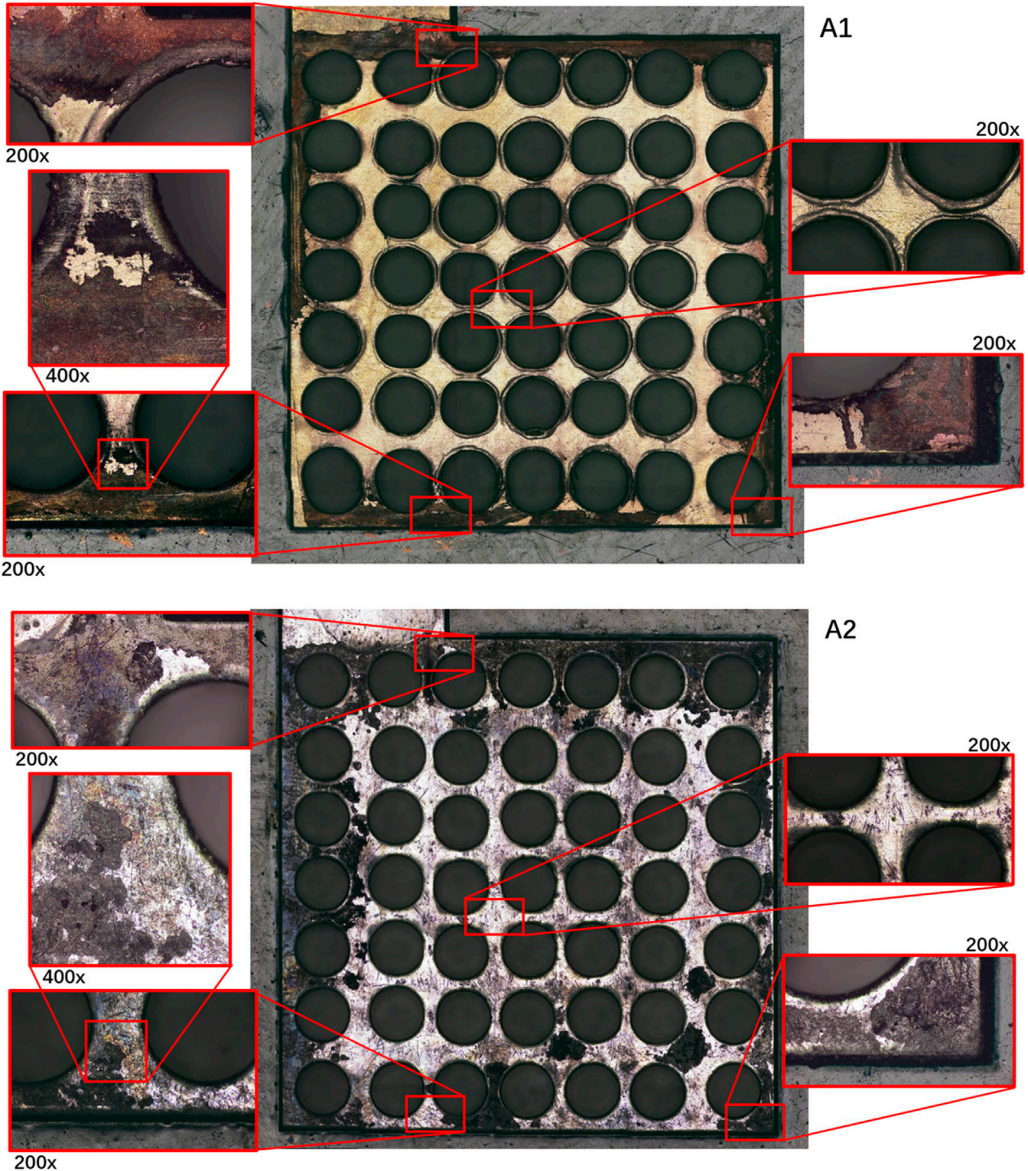
Comparison of PCB surface corrosion with different coatings (A1 and A2).

In Group A, according to the current density data and the optical microscope photos as shown in Figures 7A1, A2 of the PCB plates after the discharging test, large areas of both Au and Sn coating detachment had occurred in multiple locations. The surfaces of each electrode plate after the reaction were, respectively, imaged and compared to discuss the optimal combination for this PCB μDMFC. The corrosion mostly occurred at the edges, while the central part remained intact. The flexibility of the PCB is considered the reason for this phenomenon. Since the screws were fixed at the corners, the packaging pressure around the edges of the MEA membrane was higher than that in the central part. Thus, the contact resistance at the edge was smaller. More current passed through the edges, and the electrochemical reactions at the edges were stronger than those at the center. After the surface corrosion, the decrease in the available conductive area on the electrode plate (resulting in an increase in internal resistance) and the possibility that metal ions might have dissolved in the solution and entered the membrane with mass transfer (Zhang et al., 2023) are considered the reasons for the continuous decline in the performance of Group A. The subsequent solution ion concentration testing experiment confirmed the accurate reason for the performance degradation.

It is worth noting that the performance of μDMFC A1 and μDMFC A2 shows an increasing gap over time, whereas the performance difference between μDMFC B1 and μDMFC B2 is relatively stable. Based on the current density data and microscope photos, this study suggests that this is caused by the PCB gold plating process. Modern PCBs are produced by multi-layer technology, in which Au is usually plated on a nickel or nickel–palladium surface (Alam et al., 2002; Kwok et al., 2004; Kim et al., 2008). Once damage occurs on the Au surface, the active metal underneath the gold layer will be severely corroded, causing the surrounding gold layer to fall off more quickly. Thus, μDMFC A1 eventually showed a shorter lifespan than μDMFC B1.

**FIGURE 8:**
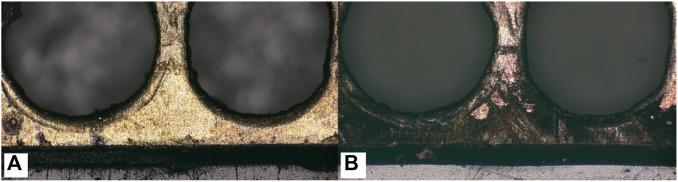
Comparing of Au layer corrosion with **(A)** and without **(B)** porous stainless-steel plate covering.

Comparing the performance of μDMFC A1 and μDMFC B1, in which both have Au-electroplated surfaces, it can be found that μDMFC B1 shows better performance than A1 in the first 2 h. The reason may be the stainless-steel porous plate, which increased the internal resistance and mass transfer resistance of μDMFC B1. However, from the 2nd hour to the end of the discharging period, μDMFC B1 began to show a higher current density than μDMFC A1. High-power optical microscope photos show that the metal layer on the surface of the PCB does not have obvious corrosion (Figure 8).

There may be two reasons for the different corrosion results. One possible reason is that during the reaction process, the isolation of the porous stainless-steel plate reduced the impact of bubbles generated by the reaction on the surface of the electrode plate, which, to some extent, slowed down the corrosion of the electrode plate and delayed the degradation of battery performance. However, through extensive literature research (Abouel-Kasem et al., 2009; Karrab et al., 2012; Song et al., 2014), it has been found that there is a significant difference in the corrosion morphology between microbubble impact and the electrode surface in this experiment. Another possible reason is that the insertion of the stainless-steel plate created a new contact corrosion potential, which mainly concentrated the electrochemical corrosion at the interface between the stainless-steel plate and the solution. Compared to the electrode plates, stainless-steel plates have stronger corrosion resistance, thus protecting the PCB coating from corrosion. Subsequently, this inference was proven by the Tafel curve test of the system.

**FIGURE 9:**
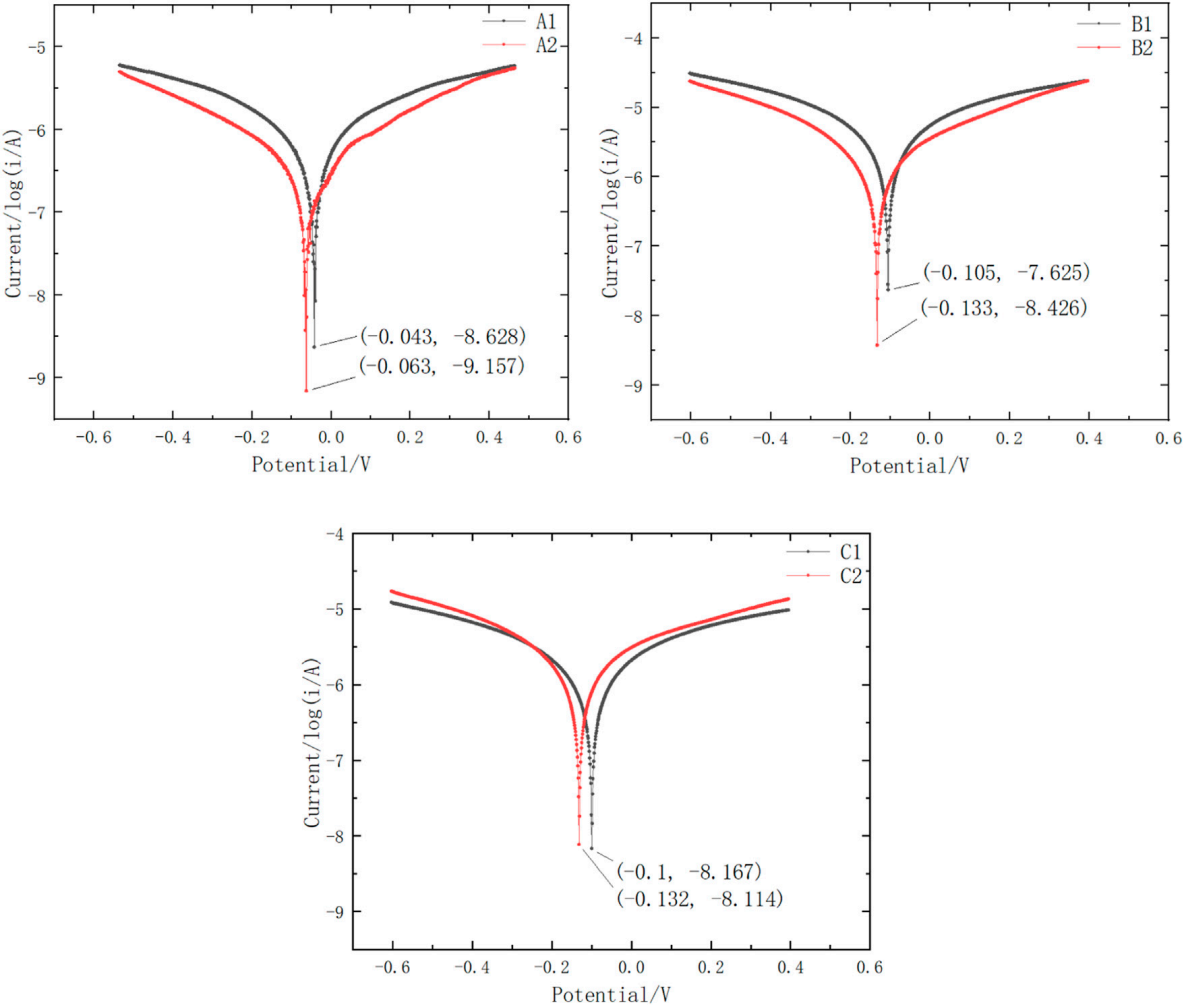
Tafel curves of different configuration current collectors.

The Tafel test is a testing method to determine the corrosion rate of metals. In order to characterize the corrosion behavior of different electrode structures more intuitively, Tafel curve tests were conducted, and the results are shown in the Figure 9. It can be seen that the corrosion potential of the gold-plated electrode plate (used in μDMFC A1) shifts forward and the corrosion current decreases, indicating an increase in the corrosion resistance performance. After adding stainless-steel porous plates, contact corrosion occurs between iron and tin/gold. Since the corrosion potential of iron is much lower than that of tin/gold, the corrosion potential of the composite system formed by the contact of the two shifts negatively, and the corrosion current increases. However, in the operating environment of DMFCs, the potential applied to the composite system is higher than the corrosion potential of both, which leads to passivation behavior on the iron surface and a decrease in corrosion current. Therefore, the corrosion behavior of the PCB surface in the μDMFC environment is suppressed.

The comprehensive comparison of A1B1 and A2B2 reveals that the porous plate has a significant effect on slowing down surface corrosion. A Sn-surfaced PCB plate, such as the plate used in μDMFC B2, when combined with a porous plate, can achieve similar performance and lifespan as an Au-electroplated PCB, such as those used in μDMFC B1.

After the 48-h discharging test, elements in the rest of the methanol solution of the eight-series solution were tested using a liquid chromatography spectrometer to explore whether the ions released after corrosion of the electrode contributed to performance degradation (As shown in Table 2).

**TABLE 2:** Ion concentration of the solution in each μDMFC after discharging.

	Cu(ug/mL)	Ni(ug/mL)	Sn(ug/mL)	Pt (ug/mL)
μDMFC A1	2.226	13.89	0.168	0.198
μDMFC A2	0.042	0.276	ND	0.462
μDMFC B1	ND	3.894	ND	0.192
μDMFC B2	ND	0.078	ND	0.216
μDMFC C1	ND	4.32	0.174	0.012
μDMFC C2	0.084	0.294	ND	0.108

(ND: not detected).

Consistent with the results obtained from the microscope photos, the ion concentration of the plate with the Au-coated anti-corrosion process is the highest, which shows that the gold-plated electrode plates suffered more severe corrosion in the later period. The ion concentration test results of Group A show that more Cu, Ni, and Sn elements were released into the solution by μDMFC A1 (Au-electroplated surface plate) compared to μDMFC A2 (Sn surface plate).

**FIGURE 10:**
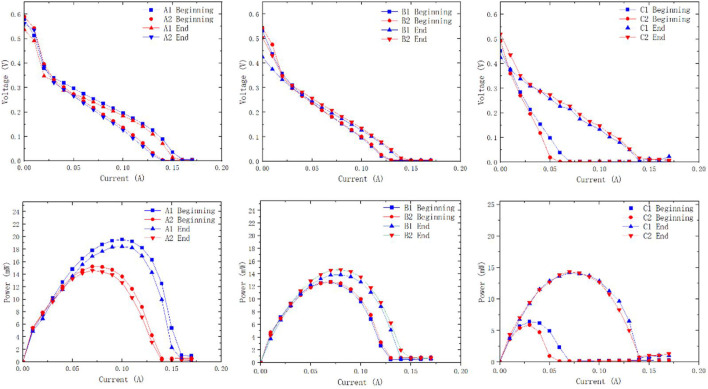
Polarization and power density curves of different configurations of µDMFCs at the beginning and the end of discharging.

After the 48-h discharging test, the MEA membrane in each μDMFC was taken out and placed in a normal plate battery for performance testing. A performance comparison of the MEA membrane before and after the discharging test was obtained (Figure 10).

Polarization curves before and after the discharging test support that the difference in membrane performance before and after the reaction is negligible. The metal ions released by the PCB plates are not sufficient to affect the performance of the membrane. It indicates that during the 48-h discharging test, the elements released by plate corrosion are not the main cause of performance degradation. The decrease in the available surface area and increase in internal resistance caused by plate corrosion are considered the main influencing factors.

Based on Group B, the configuration of the hollowed-out PCB plates was tested in Group C, which significantly reduced the PCB area, in order to explore the impact of metal ions released by the PCB electrode on the performance of μDMFCs. Meanwhile, if this structure shows excellent performance, the manufacturing cost of PCB plates can be significantly reduced. The experimental results indicate that the performance of Group C is lower than that of Group B. The reason is likely due to insufficient packaging pressure, resulting in the mass transfer obstructed by poor contact (Zhenyu et al., 2012). The strong uniform pressure between the electrode plate and the membrane is one of the most important factors affecting the performance of μDMFCs. Due to the weaker rigidity of PCB electrode materials compared to metal electrode plates, Group C with hollowed-out PCB plates results in a slight protrusion in the center of the stainless-steel plate. In this type of package, certain areas or parts of the current collectors cannot reach the target packaging pressure, which results in performance loss.

**FIGURE 11:**
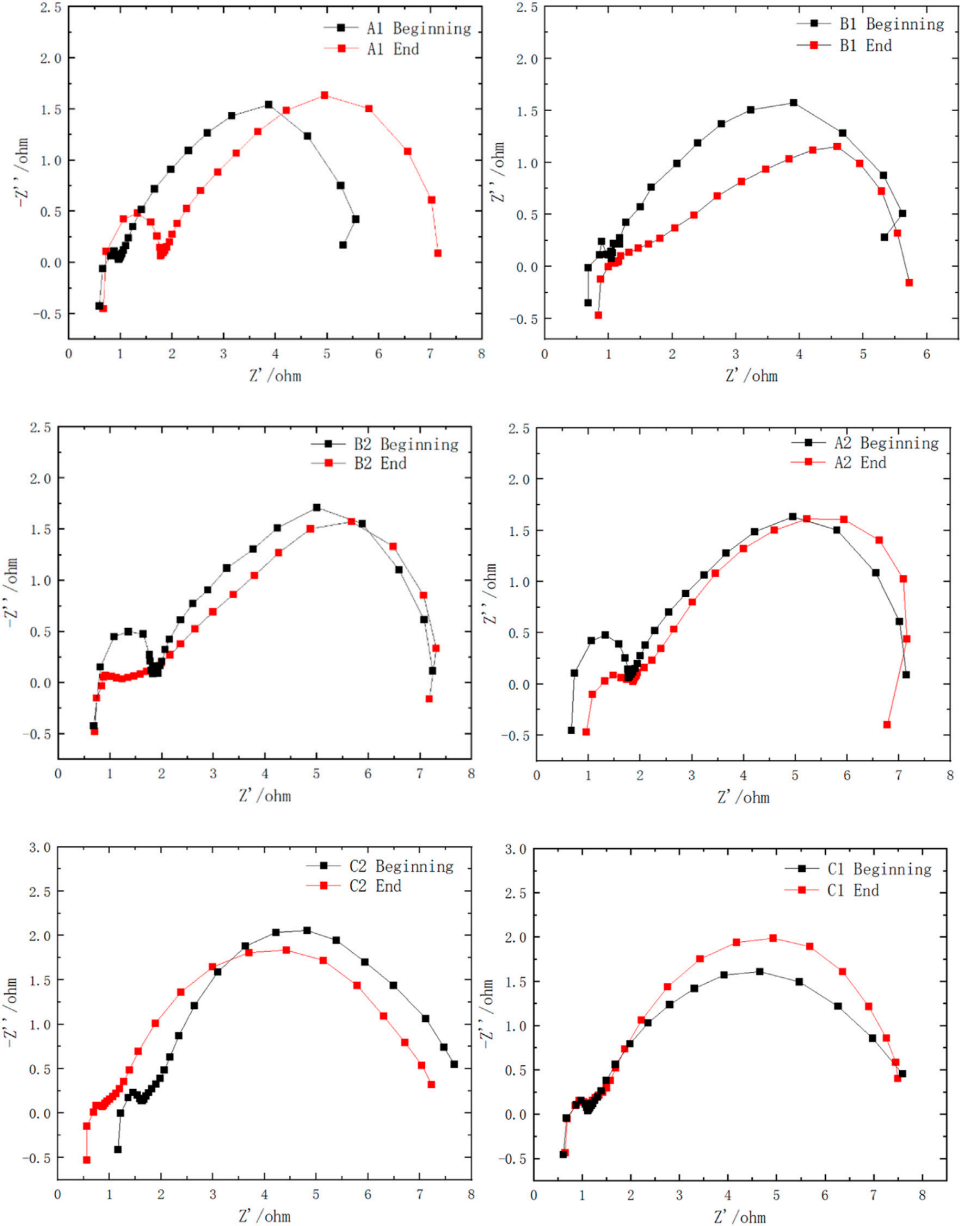
EIS of different configurations of µDMFCs at the beginning and end of 0.2 V discharging.

To further elaborate the effect of the novel structure on the MEA performance, electrochemical impedance spectrum (EIS) measurements at a discharging current density of 20 mA cm^-2^ with a 2.0 M methanol solution were carried out at 333 K, and the results are shown in Figure 11. It was pointed out that the right intercept with the real coordinate axis on the Nyquist plot (low frequency data) is related to the charge transfer resistance (CTR) of the electrode reaction. A high CTR indicates a large reaction resistance and a strong polarization of the electrode process. The inductive loop in the lowest frequency range arises from the absorption/desorption of CO as a result of the methanol oxidation reaction (Jens and Urban, 1999).

The impedance spectra of the full cell reveal that at the very beginning, Au-coated current collectors have a slightly increased inner resistance compared with the Sn-coated current collectors, which is expressed as a more left intercept with the real coordinate axis in the high-frequency range. After a long discharging test, the Au- and Sn-coated groups show increased internal resistance as a result of coating corrosion. In the low frequency range, the impedance spectra of the groups with the porous plate show a smaller gap with the beginning of discharging, confirming that the addition of the porous plate reduced the performance degradation successfully.

## 4 Conclusion

The work in this paper conjectured and verified the performance degradation factors of PCB current collectors in µDMFCs by testing µDMFCs with different designed configurations. The experiments showed that all types of PCB coating can benefit from the porous stainless-steel plates covering to a great extent. At the end of 48 h of discharging, µDMFCs with porous stainless-steel plates between MEA and PCB coating achieved higher performance than that of the direct contacting series.

The conclusions of this work explore a practical direction to enhance the cost-effectiveness of fuel cells, thereby promoting the large-scale application of DMFCs. The integration of PCB technology with the current collectors makes these novel μDMFCs more accessible for use in electrical devices. Particularly, when working in an environment with a higher temperature, μDMFCs can realize their full potential. As long as the production line is well-equipped and the fabrication cost can be reduced to a commercial level, this type of µDMFCs will be a highly efficient and clean portable power resource in the future.

## Data Availability

The original contributions presented in the study are included in the article/Supplementary Material; further inquiries can be directed to the corresponding authors.
